# Supplementary material for: A Likelihood Approach for Real-Time Calibration of Stochastic Compartmental Epidemic Models
Source: PLoS Comput Biol. 2017 Jan 17;13(1):e1005257. doi: 10.1371/journal.pcbi.1005257 (PMC5240920; doi:10.1371/journal.pcbi.1005257)
Supplement: S1 File — (TAR.GZ) [file pcbi.1005257.s014.tar.gz › HSPH_Online-SI-Revision/Additions/IPoi10/n100-extreme-SEITR_noisy/n100-extreme-SEITR_noisy_BENCH2_table-4.pdf]

1	newly 3 weeks spec	13577.1	7620.81	{3782., 9631.}	43.8699	0.301314	newly 3 weeks cumul	31325.2	19389.2	{7341., 27860.}	38.1034	0.280993	newly 5 weeks spec	12047.4	8220.19	{6591., 9690.}	31.7677	0.18292	newly 5 weeks cumul	57070.8	35782.4	{20271., 44229.}	37.3016	0.240147	newly 54 weeks cumul	86564.8	82391.	{71588., 90984.}	7.46876	0.034774
2	newly 3 weeks spec	17235.	4323.07	{882., 7393.}	74.9169	0.735893	newly 3 weeks cumul	29177.	7402.28	{1430., 13030.}	74.6298	0.743401	newly 5 weeks spec	17453.	10537.	{4586., 13797.}	39.6263	0.277254	newly 5 weeks cumul	67910.	25400.6	{7983., 38398.}	62.5967	0.51455	newly 62 weeks cumul	97717.	91791.2	{91253., 92370.}	6.06422	0.0271773
3	newly 3 weeks spec	14424.	8492.02	{1886., 15224.}	44.5883	0.37279	newly 3 weeks cumul	24222.	14586.2	{2692., 28061.}	49.5848	0.413782	newly 5 weeks spec	18266.	14727.1	{10854., 17239.}	19.3744	0.110074	newly 5 weeks cumul	61841.	41994.9	{18934., 60616.}	32.9336	0.22276	newly 64 weeks cumul	97783.	92634.7	{91309., 93524.}	5.26501	0.0235225
4	newly 3 weeks spec	13822.	11656.5	{4239., 15638.}	26.7523	0.247821	newly 3 weeks cumul	25848.	24966.7	{6589., 40527.}	42.6355	0.322119	newly 5 weeks spec	16022.	12477.4	{9049., 15658.}	22.1236	0.186024	newly 5 weeks cumul	59339.	50487.8	{28581., 63338.}	18.7626	0.190073	newly 62 weeks cumul	94062.	85735.	{81948., 90471.}	8.85262	0.122652
5	newly 3 weeks spec	22578.	5761.83	{1406., 11319.}	74.4804	0.742924	newly 3 weeks cumul	36477.	9234.74	{2077., 19451.}	74.6834	0.76136	newly 5 weeks spec	16920.	13789.9	{9070., 16390.}	18.4993	0.132775	newly 5 weeks cumul	77798.	33347.1	{15248., 50912.}	57.1363	0.443376	newly 64 weeks cumul	97923.	78914.6	{75595., 90987.}	19.4116	0.094685
6	newly 3 weeks spec	20143.	6061.75	{639., 11071.}	69.9064	0.690744	newly 3 weeks cumul	34123.	10003.1	{995., 19060.}	70.6852	0.719263	newly 5 weeks spec	17405.	13202.9	{4379., 16238.}	24.143	0.176296	newly 5 weeks cumul	74866.	33413.4	{6863., 50441.}	55.3691	0.444791	newly 63 weeks cumul	93450.	88567.3	{86164., 93788.}	5.3447	0.0240763
7	newly 3 weeks spec	12108.	8731.02	{5179., 10305.}	27.8905	0.160124	newly 3 weeks cumul	28975.	22122.6	{10457., 29371.}	24.2716	0.152214	newly 5 weeks spec	11332.	8978.28	{7293., 10193.}	20.7706	0.106605	newly 5 weeks cumul	52923.	40321.3	{27356., 46124.}	23.8114	0.129953	newly 55 weeks cumul	88800.	80664.6	{70533., 88612.}	9.29816	0.0442402
8	newly 3 weeks spec	16980.	6206.9	{1152., 11809.}	63.4458	0.583115	newly 3 weeks cumul	32632.	10788.4	{1736., 22003.}	66.9393	0.650341	newly 5 weeks spec	14766.	12203.7	{6477., 15218.}	18.799	0.126058	newly 5 weeks cumul	65812.	32638.5	{11096., 51501.}	50.4065	0.382179	newly 62 weeks cumul	97109.	92225.8	{90982., 93021.}	5.0286	0.0224253
9	newly 3 weeks spec	23304.	2037.64	{163., 4626.}	91.2563	1.42523	newly 3 weeks cumul	35304.	3124.31	{231., 7123.}	91.1503	1.40942	newly 5 weeks spec	17411.	8176.1	{1556., 14901.}	53.0406	0.615005	newly 5 weeks cumul	78660.	15830.7	{2276., 31758.}	79.8745	1.00363	newly 64 weeks cumul	98833.	87256.	{88859., 92522.}	11.7137	0.267683
10	newly 3 weeks spec	12030.	11952.	{9969., 13376.}	12.2716	0.0643273	newly 3 weeks cumul	25011.	30977.3	{19068., 37572.}	37.486	0.156284	newly 5 weeks spec	13614.	10107.1	{7387., 13149.}	25.7595	0.138757	newly 5 weeks cumul	52825.	52664.7	{44865., 57295.}	9.59403	0.0477175	newly 60 weeks cumul	95503.	83623.3	{75822., 91670.}	12.4391	0.0591845
11	newly 3 weeks spec	13896.	5732.83	{1787., 8924.}	58.7447	0.46079	newly 3 weeks cumul	26326.	11563.4	{3202., 18668.}	56.3246	0.452479	newly 5 weeks spec	14675.	9447.68	{5735., 11261.}	35.6206	0.213197	newly 5 weeks cumul	57151.	28872.4	{12287., 40706.}	49.4806	0.345144	newly 59 weeks cumul	97210.	91689.9	{90006., 93343.}	5.67855	0.0254804
12	newly 3 weeks spec	16108.	8302.22	{2421., 13044.}	48.4591	0.371078	newly 3 weeks cumul	33166.	16748.4	{4065., 30031.}	49.5191	0.405314	newly 5 weeks spec	14159.	11733.1	{8672., 13393.}	17.1333	0.0992446	newly 5 weeks cumul	64453.	39110.9	{17770., 54478.}	39.3187	0.263405	newly 60 weeks cumul	95054.	90497.9	{86758., 92639.}	4.79316	0.0214553
13	newly 3 weeks spec	15592.	9997.36	{3960., 13761.}	35.8815	0.241117	newly 3 weeks cumul	30732.	20568.7	{6385., 32268.}	35.8544	0.254951	newly 5 weeks spec	14704.	12454.	{10684., 13852.}	15.3022	0.077832	newly 5 weeks cumul	62511.	45158.3	{26042., 57047.}	27.7595	0.164512	newly 61 weeks cumul	96828.	89275.4	{85927., 91996.}	7.80006	0.0354591
14	newly 3 weeks spec	10081.	7963.94	{5033., 9338.}	21.0005	0.140166	newly 3 weeks cumul	23444.	22652.5	{10272., 27119.}	18.8525	0.129471	newly 5 weeks spec	11642.	7094.33	{5165., 8861.}	39.0626	0.235789	newly 5 weeks cumul	46832.	37483.	{25805., 42821.}	19.9628	0.125723	newly 54 weeks cumul	89533.	70031.8	{54556., 85274.}	21.781	0.112222
15	newly 3 weeks spec	14236.	7109.98	{3204., 9560.}	50.0563	0.354802	newly 3 weeks cumul	30841.	17221.6	{6118., 26534.}	44.16	0.329746	newly 5 weeks spec	13230.	8379.03	{6597., 9608.}	36.6664	0.213603	newly 5 weeks cumul	59486.	33609.	{18023., 43604.}	43.5011	0.286664	newly 55 weeks cumul	91749.	84788.5	{76696., 91159.}	7.60595	0.0355566
16	newly 3 weeks spec	11254.	10044.1	{6559., 11726.}	13.2825	0.0766605	newly 3 weeks cumul	24092.	28494.	{12463., 34232.}	35.6133	0.159972	newly 5 weeks spec	12953.	8269.76	{5364., 11518.}	36.1557	0.2099	newly 5 weeks cumul	50447.	46171.3	{33751., 51845.}	10.1141	0.0551371	newly 58 weeks cumul	87287.	73811.6	{58869., 88266.}	15.8947	0.0793722
17	newly 3 weeks spec	15615.	3763.76	{384., 8171.}	75.8965	0.923938	newly 3 weeks cumul	24517.	5843.21	{559., 12936.}	76.1667	0.945208	newly 5 weeks spec	20574.	11440.2	{2877., 16706.}	44.3948	0.439774	newly 5 weeks cumul	68260.	24764.6	{4589., 43482.}	63.7201	0.671309	newly 64 weeks cumul	92364.	90020.2	{90630., 93300.}	2.87632	0.100321
18	newly 3 weeks spec	19007.	3236.61	{531., 7107.}	82.9715	0.98748	newly 3 weeks cumul	31170.	5318.02	{804., 11848.}	82.9387	1.00054	newly 5 weeks spec	17189.	9147.05	{3271., 14560.}	46.7854	0.378624	newly 5 weeks cumul	70235.	20418.1	{5445., 38241.}	70.9289	0.681731	newly 63 weeks cumul	98793.	90632.4	{88743., 93578.}	8.26026	0.0375497
19	newly 3 weeks spec	12889.	4169.36	{339., 8679.}	67.6518	0.699431	newly 3 weeks cumul	24953.	8447.86	{608., 19165.}	66.1449	0.704014	newly 5 weeks spec	14542.	7284.83	{1458., 10668.}	49.9049	0.407194	newly 5 weeks cumul	54787.	21525.1	{2754., 40197.}	60.7112	0.562924	newly 58 weeks cumul	96606.	92110.4	{89204., 93778.}	4.65355	0.0207765
20	newly 3 weeks spec	19368.	5328.89	{843., 11004.}	72.4861	0.735349	newly 3 weeks cumul	33454.	8694.56	{1195., 18964.}	74.0104	0.777444	newly 5 weeks spec	16864.	12751.	{5749., 16242.}	24.3895	0.178279	newly 5 weeks cumul	72041.	30732.6	{9324., 50328.}	57.3402	0.46474	newly 63 weeks cumul	97397.	92023.1	{90834., 92718.}	5.51751	0.0246632
21	newly 3 weeks spec	19672.	2807.37	{393., 5874.}	85.7291	1.03487	newly 3 weeks cumul	30439.	4231.18	{560., 9058.}	86.0995	1.05356	newly 5 weeks spec	18543.	10661.2	{2871., 16362.}	42.5055	0.323449	newly 5 weeks cumul	73114.	21103.4	{4467., 37469.}	71.1363	0.654354	newly 64 weeks cumul	98246.	91939.7	{91029., 93454.}	6.41888	0.0288383
22	newly 3 weeks spec	14910.	12066.8	{9843., 13638.}	19.0692	0.098535	newly 3 weeks cumul	31688.	33152.3	{20545., 38882.}	20.9189	0.100063	newly 5 weeks spec	14003.	9397.22	{5979., 13071.}	32.8914	0.190147	newly 5 weeks cumul	61831.	53675.8	{46764., 58031.}	13.1895	0.0646542	newly 60 weeks cumul	92206.	78921.	{64837., 90181.}	14.4079	0.0704606
23	newly 3 weeks spec	16317.	11139.8	{4752., 15109.}	31.7286	0.229054	newly 3 weeks cumul	34910.	22314.	{7601., 34395.}	37.0624	0.283463	newly 5 weeks spec	13260.	13202.3	{11084., 15084.}	12.0743	0.0626228	newly 5 weeks cumul	65072.	48883.8	{30229., 60861.}	24.8774	0.15671	newly 61 weeks cumul	83725.	89164.4	{85954., 92633.}	6.62644	0.0276969
24	newly 3 weeks spec	16757.	1844.66	{120., 4131.}	88.9917	1.23906	newly 3 weeks cumul	25641.	3028.81	{193., 6848.}	88.1876	1.21796	newly 5 weeks spec	20012.	6241.91	{739., 11903.}	68.8092	0.670165	newly 5 weeks cumul	69280.	12931.6	{1192., 26473.}	81.3343	0.933889	newly 63 weeks cumul	98228.	92232.1	{91797., 92513.}	6.10405	0.0273556
25	newly 3 weeks spec	12372.	6366.98	{1877., 10782.}	48.5371	0.431683	newly 3 weeks cumul	23919.	13022.8	{3200., 24625.}	48.2642	0.431427	newly 5 weeks spec	15457.	9898.77	{6539., 11945.}	35.9593	0.253332	newly 5 weeks cumul	55191.	31396.	{13492., 47689.}	43.1138	0.339421	newly 59 weeks cumul	91517.	87808.	{85069., 90068.}	4.0528	0.0181193
26	newly 3 weeks spec	12283.	8350.88	{2189., 11648.}	32.0127	0.274874	newly 3 weeks cumul	28429.	19761.8	{3738., 31699.}	35.8684	0.321665	newly 5 weeks spec	11730.	9452.78	{6776., 11540.}	19.4247	0.129253	newly 5 weeks cumul	53475.	38492.8	{14607., 51258.}	28.0172	0.217318	newly 57 weeks cumul	80102.	86983.7	{79521., 92741.}	9.42069	0.0387188
27	newly 3 weeks spec	12409.	9119.79	{5892., 11566.}	26.5067	0.164461	newly 3 weeks cumul	28757.	26793.8	{11109., 33288.}	19.9201	0.133057	newly 5 weeks spec	11908.	7474.98	{4195., 10989.}	37.2272	0.227889	newly 5 weeks cumul	53686.	42721.4	{31028., 50391.}	20.4237	0.118674	newly 57 weeks cumul	90968.	69697.1	{47641., 87838.}	23.3829	0.124878
28	newly 3 weeks spec	16959.	12392.4	{4347., 17916.}	29.4385	0.252215	newly 3 weeks cumul	28083.	22585.4	{6206., 36290.}	38.0169	0.296739	newly 5 weeks spec	18063.	14709.3	{9838., 18066.}	18.9137	0.11994	newly 5 weeks cumul	68202.	52789.8	{33364., 65798.}	22.5992	0.161677	newly 64 weeks cumul	88415.	86528.9	{81704., 91860.}	4.83154	0.0214521
29	newly 3 weeks spec	12914.	11370.	{6636., 13844.}	17.7651	0.150632	newly 3 weeks cumul	24569.	27029.4	{11561., 37766.}	38.9681	0.231949	newly 5 weeks spec	15388.	10781.7	{8500., 13635.}	29.9344	0.178574	newly 5 weeks cumul	56007.	49582.3	{35223., 58466.}	14.6763	0.11225	newly 61 weeks cumul	96532.	86056.4	{80079., 91361.}	10.852	0.0505607
30	newly 3 weeks spec	13125.	2563.58	{359., 5549.}	80.468	0.920014	newly 3 weeks cumul	20142.	3926.17	{557., 8501.}	80.5075	0.929188	newly 5 weeks spec	19690.	9788.38	{2929., 15447.}	50.2876	0.419215	newly 5 weeks cumul	60177.	19343.2	{4543., 34727.}	67.8562	0.638517	newly 64 weeks cumul	98710.	92313.	{92050., 92578.}	6.48063	0.0291013
31	newly 3 weeks spec	10370.	9094.87	{6948., 10265.}	12.3187	0.0623186	newly 3 weeks cumul	26082.	25698.6	{19486., 29477.}	13.2088	0.064324	newly 5 weeks spec	9931.	7841.84	{5336., 10010.}	21.5289	0.118391	newly 5 weeks cumul	46868.	42277.9	{34779., 46282.}	9.7937	0.0480043	newly 54 weeks cumul	76589.	72284.	{56040., 85733.}	12.752	0.0609194
32	newly 3 weeks spec	12614.	11413.9	{7333., 13922.}	18.0011	0.139123	newly 3 weeks cumul	26998.	26658.6	{12839., 38673.}	32.7939	0.208084	newly 5 weeks spec	13001.	11276.8	{8251., 13992.}	17.255	0.107906	newly 5 weeks cumul	54036.	50057.6	{38228., 58556.}	14.2584	0.10362	newly 60 weeks cumul	93822.	86059.	{78565., 91122.}	8.27414	0.038357
33	newly 3 weeks spec	20264.	17179.8	{13779., 19670.}	15.2338	0.0795925	newly 3 weeks cumul	37971.	42851.8	{26385., 52678.}	28.1642	0.122576	newly 5 weeks spec	15166.	10677.9	{5925., 16543.}	34.0098	0.205831	newly 5 weeks cumul	73450.	68374.9	{61817., 72669.}	6.90966	0.0330272	newly 64 weeks cumul	96763.	85097.4	{76468., 92171.}	12.0558	0.0574486
34	newly 3 weeks spec	15602.	11365.9	{5531., 13980.}	27.151	0.180091	newly 3 weeks cumul	34185.	26622.9	{9278., 38317.}	28.5592	0.204349	newly 5 weeks spec	13645.	11306.8	{8308., 13894.}	17.8299	0.0972025	newly 5 weeks cumul	63916.	50026.	{31762., 58820.}	21.7317	0.130789	newly 60 weeks cumul	89965.	86250.1	{80018., 91717.}	4.95481	0.0225643
35	newly 3 weeks spec	18683.	3228.84	{227., 6569.}	82.7178	1.07555	newly 3 weeks cumul	29482.	4952.36	{329., 10228.}	83.2021	1.08681	newly 5 weeks spec	19217.	11174.5	{2103., 16275.}	41.851	0.47037	newly 5 weeks cumul	73185.	22969.	{3153., 39441.}	68.6151	0.761209	newly 64 weeks cumul	94833.	88007.1	{90702., 91572.}	7.19786	0.211667
36	newly 3 weeks spec	11929.	3676.27	{284., 7465.}	69.1821	0.718215	newly 3 weeks cumul	21930.	6824.	{490., 14512.}	68.8828	0.730389	newly 5 weeks spec	15024.	7959.42	{1180., 11787.}	47.0219	0.402246	newly 5 weeks cumul	52068.	20578.7	{2263., 36587.}	60.4772	0.565901	newly 60 weeks cumul	97587.	89325.2	{88003., 90193.}	8.46606	0.0384384
37	newly 3 weeks spec	15965.	1697.38	{110., 3473.}	89.3681	1.16873	newly 3 weeks cumul	25084.	3022.48	{200., 6357.}	87.9506	1.12317	newly 5 weeks spec	18951.	4977.15	{529., 8826.}	73.7367	0.708684	newly 5 weeks cumul	65780.	11072.9	{922., 21120.}	83.1667	0.928997	newly 61 weeks cumul	98650.	88963.9	{88081., 89148.}	9.81862	0.0449307
38	newly 3 weeks spec	14686.	9922.29	{5847., 11865.}	32.4371	0.274822	newly 3 weeks cumul	31691.	24660.6	{11009., 33767.}	25.3546	0.225673	newly 5 weeks spec	13535.	9671.02	{7323., 11689.}	28.5481	0.208408	newly 5 weeks cumul	60924.	44669.5	{31015., 52027.}	26.6799	0.208206	newly 58 weeks cumul	87770.	81387.6	{71554., 88590.}	7.78328	0.0365194
39	newly 3 weeks spec	14352.	10426.8	{4744., 13281.}	27.3494	0.20712	newly 3 weeks cumul	28679.	23460.5	{7818., 35676.}	29.8471	0.227495	newly 5 weeks spec	15042.	11264.9	{8298., 13352.}	25.1101	0.146011	newly 5 weeks cumul	60279.	46299.	{27998., 56793.}	23.1922	0.157379	newly 60 weeks cumul	93593.	88955.9	{82289., 93357.}	5.00502	0.0227651
40	newly 3 weeks spec	14498.	13077.6	{10719., 15182.}	11.6639	0.0613904	newly 3 weeks cumul	31327.	35486.6	{21899., 42142.}	28.9503	0.128144	newly 5 weeks spec	13194.	9774.63	{6370., 14590.}	29.912	0.168187	newly 5 weeks cumul	59514.	57241.8	{50013., 62135.}	7.16407	0.0351486	newly 61 weeks cumul	94658.	80788.3	{69434., 91073.}	14.6525	0.0714843
41	newly 3 weeks spec	13435.	866.627	{81., 2184.}	93.5495	1.47925	newly 3 weeks cumul	19619.	1346.96	{126., 3457.}	93.1344	1.45815	newly 5 weeks spec	23349.	4102.45	{519., 9106.}	82.4299	0.986902	newly 5 weeks cumul	66595.	7459.34	{868., 17414.}	88.7989	1.20814	newly 64 weeks cumul	95882.	91523.1	{92677., 93090.}	4.54609	0.102752
42	newly 3 weeks spec	10315.	8771.71	{6533., 10264.}	15.1535	0.116957	newly 3 weeks cumul	25161.	23667.8	{13357., 29680.}	21.9129	0.156261	newly 5 weeks spec	10541.	8134.9	{5643., 10164.}	22.8261	0.133326	newly 5 weeks cumul	46907.	40533.6	{31619., 46450.}	13.5877	0.0987101	newly 55 weeks cumul	87719.	77363.	{63638., 87866.}	12.3172	0.0596739
43	newly 3 weeks spec	12045.	10898.9	{5884., 13213.}	16.8411	0.12713	newly 3 weeks cumul	24152.	28479.7	{10410., 37292.}	42.4549	0.223096	newly 5 weeks spec	14237.	9547.53	{6225., 12936.}	32.9386	0.197375	newly 5 weeks cumul	52694.	48727.8	{32472., 56719.}	12.827	0.0898949	newly 60 weeks cumul	95113.	79986.7	{65940., 89803.}	15.9035	0.0777681
44	newly 3 weeks spec	16196.	7185.39	{1490., 12191.}	55.6348	0.503784	newly 3 weeks cumul	32797.	14453.2	{2376., 26624.}	56.0623	0.538757	newly 5 weeks spec	14170.	10694.7	{6014., 13210.}	24.5255	0.173404	newly 5 weeks cumul	64055.	34551.1	{11486., 51697.}	46.0603	0.364415	newly 60 weeks cumul	95957.	89568.	{86906., 91763.}	6.65822	0.0300655
45	newly 3 weeks spec	16795.	5824.51	{777., 12015.}	65.32	0.678982	newly 3 weeks cumul	31747.	10078.6	{1172., 22356.}	68.2535	0.737788	newly 5 weeks spec	14996.	11821.9	{4599., 15282.}	21.8751	0.211688	newly 5 weeks cumul	65154.	31111.4	{7780., 51562.}	52.2494	0.467703	newly 62 weeks cumul	96985.	92284.8	{91026., 93086.}	4.84632	0.0215907
46	newly 3 weeks spec	15111.	13368.8	{4877., 17180.}	21.6024	0.137396	newly 3 weeks cumul	25292.	26348.7	{7219., 40193.}	37.6923	0.198611	newly 5 weeks spec	17991.	14313.4	{11294., 16977.}	20.4411	0.104706	newly 5 weeks cumul	63693.	56071.2	{33878., 67014.}	14.2817	0.0799125	newly 64 weeks cumul	97957.	91107.5	{88159., 93251.}	6.99234	0.0315968
47	newly 3 weeks spec	13356.	9487.46	{3352., 12278.}	28.9648	0.196037	newly 3 weeks cumul	27095.	22028.8	{5924., 32652.}	30.5795	0.210472	newly 5 weeks spec	14547.	10473.9	{8329., 12191.}	27.9994	0.148499	newly 5 weeks cumul	57180.	43055.3	{21355., 53252.}	24.7022	0.149808	newly 59 weeks cumul	92154.	84210.7	{78554., 89461.}	8.61962	0.039715
48	newly 3 weeks spec	14508.	8688.56	{1617., 13955.}	40.1119	0.328113	newly 3 weeks cumul	25267.	17042.	{2576., 30831.}	43.4694	0.350943	newly 5 weeks spec	17877.	12315.6	{7693., 14468.}	31.109	0.179091	newly 5 weeks cumul	62499.	40584.8	{14299., 57337.}	35.0632	0.240924	newly 62 weeks cumul	95356.	91467.4	{89011., 93501.}	4.078	0.0181999
49	newly 3 weeks spec	15110.	12185.2	{9949., 14438.}	19.3568	0.101148	newly 3 weeks cumul	34829.	37535.8	{33718., 40639.}	14.5174	0.0687935	newly 5 weeks spec	12499.	7787.91	{5223., 11232.}	38.6695	0.227087	newly 5 weeks cumul	62570.	55415.6	{49462., 60555.}	11.4342	0.0561365	newly 60 weeks cumul	88106.	74089.8	{60334., 87141.}	16.4584	0.0814108
50	newly 3 weeks spec	13796.	11445.4	{6643., 14435.}	19.5728	0.128274	newly 3 weeks cumul	28249.	24568.4	{11038., 37782.}	29.5543	0.18354	newly 5 weeks spec	14245.	12542.6	{9357., 14556.}	12.7739	0.0647303	newly 5 weeks cumul	58206.	50099.6	{36035., 60670.}	15.4777	0.0894714	newly 61 weeks cumul	94140.	89700.4	{84728., 92818.}	4.71597	0.0212418
